# Tolerability and safety of urotainer® polihexanide 0.02% in catheterized patients: a prospective cohort study

**DOI:** 10.1186/s12894-020-00650-1

**Published:** 2020-07-08

**Authors:** Jürgen Pannek, Karel Everaert, Sandra Möhr, Will Vance, Frank Van der Aa, Jürg Kesselring

**Affiliations:** 1 Neuro-Urology, Swiss Paraplegic Centre, Guido A. Zäch Strasse 1, CH – 6207 Nottwil, Switzerland; 2 Department of Urology, Inselspital, Bern University Hospital, University of Bern, Bern, Switzerland; 3 Urologie, Universitair Ziekenhuis, Gent, Belgium; 4 Schweizerisches Paraplegikerzentrum REHAB, Basel, Switzerland; 5 Urology, Kliniken Beelitz GmbH, Beelitz-Heilstätten, Beelitz, Germany; 6 National Multiple Sclerose Centrum, Melsbroek, Belgium; 7 Department of Neurology & Neurorehabilitation, Kliniken Valens, Valens, Switzerland

**Keywords:** Urinary tract infection, Indwelling catheter, Polyhexanide, Bladder irrigation

## Abstract

**Background:**

In patients with indwelling bladder catheters for > 2 weeks, bacterial colonization is inevitable, leading to urinary tract infections or encrustations with subsequent catheter blockage. Currently, bladder irrigations are the most frequently used prophylactic means, but the best solution remains yet to be determined. In vitro studies demonstrate that polihexanide is a promising option for catheter irrigation, but no data about safety and tolerability exist.

**Methods:**

In a prospective observational study in patients with indwelling bladder catheter for > 2 weeks, a 0.02% polihexanide solution was used to rinse the catheter on five consecutive days. Adverse events, tolerability and vital signs were assessed before, during, after and at the end of the treatment period.

**Results:**

There was no serious adverse event in the study. A total of 28 adverse events (AEs) in 15 (46.88%) participants were experienced. Absolute changes in pain scores were not clinically relevant. No incidences of either flushing or sweating were found during instillation. Bladder spasms during instillation were reported in two cases during a single instillation. Mean pulse rates did not change by more than 3 beats per minute. Mean changes in body temperature did not exceed 0.12 °C. Clinically relevant changes in blood pressure were recorded for 3 patients.

**Conclusions:**

This is the first study to demonstrate that a 0.02% polihexanide solution can safely be used for catheter irrigation.

**Trial registration:**

ClinicalTrials.gov (NCT02157415), June 6th, 2014.

## Background

Despite recent advances in urologic diagnostics and treatment, a significant group of patients has to rely on long-term indwelling urinary catheters, either due to urinary retention, urinary incontinence, neurogenic lower urinary tract dysfunction or due to impaired general health [1]. Indwelling urinary catheters, however, are associated with significant long-term risks, including, but not limited to, catheter-associated urinary tract infections (CAUTI), and catheter encrustations [2]. The latter decrease the quality of life of the affected persons, as they may lead to incontinence or retention due to blockage of the lumen of the catheter. The former are even related to a significant mortality. UTI made up the highest number of infections (> 560,000) compared to other hospital acquired infections, and attributable deaths from UTI were estimated to be over 13,000 (mortality rate 2.3%) [3]. Furthermore, CAUTI are a significant financial burden for the health system. Costs associated with the treatment of CAUTI are at least $1000, with many factors leading to costs far greater [4]. Therefore, prophylaxis of catheter-related complications are increasingly important.

Colonization of urinary catheters with microorganisms is inevitable. It is perpetuated by microorganisms-produced biofilm, made up of proteins, electrolytes, and other components of urine on the surface of the catheter [5]. About 50% of long-term catheterized patients develop such encrustations which block the lumen of the catheter [6]. As the only causal treatment would be a bladder management without an indwelling catheter, in persons requiring a catheter for long-term urinary drainage merely symptomatic treatment is possible. Among those, bladder irrigation, e.g. with citric acid containing solutions to dissolve the crystals is commonly utilized [7]. Currently, however, no strategy with evidence-proven efficacy and tolerability exists [8].

Polihexanide has been developed as an improved, second generation of chlorhexidine. It has been used for over 15 years as an antiseptic ingredient. It is a broad-spectrum biocide not only effective against bacteria, but also against certain fungal and protozoal pathogens [9]. Given its antimicrobial activity, catheter irrigations with polihexanide may be a promising tool for the prevention of CAUTI. Prior to testing its efficacy, however, tolerability and side effects have to be assessed.

Therefore, we undertook an open-label prospective observational cohort study using a polihexanide-containing medical device (Uro-Tainer® Polihexanide 0.02%; UT-PHMB) in persons with bladder catheters for > 2 weeks. The primary objective was to assess its tolerability. The secondary objective was to evaluate the safety which was assessed in terms of incidence of adverse events.

## Methods

### Device description

UT-PHMB is a ready-to-use, disposable system. It consists of a PVC-free bag containing 100 ml of sterile solution (polihexanide 0.02%, sorbitol (5 g per 100 mL) in water), a flexible tube fitted with a clamp, and a sterile universal connector. The primary intended purpose of UT-PHMB is the mechanical rinsing of the catheter (removal of debris) whereas the secondary intended purpose is the bacterial decolonization of the catheter. The CE-mark for UT-PHMB was obtained on March 27, 2013.

### Study design

A prospective open-label, multicentric, non-comparative cohort study was performed at six centers in Switzerland, Belgium, and Germany. The objectives of this study were to assess the tolerability and safety of UT-PHMB in subjects catheterized for > 2 weeks. Criteria for insufficient tolerability were: occurrence of bladder spasms as reported by the patient, change in systolic blood pressure ≥ 40 mmHg, change of heart rate ≥ 30 beats per minute, change in body temperature > 2 °C, and occurrence of flushing or sweating.

Competent Independent Ethics Committees reviewed and approved the study protocol, any protocol amendments and the patient information sheet and consent form. The informed consent, written in accordance with the origins of the Declaration of Helsinki and the applicable laws of the country had to be obtained from all patients. All patients signed the informed consent form before being enrolled into the study. The Investigator explained the nature, purpose and risks of the study and provided the patients with a copy of the patient information sheet. The patients were given sufficient time to consider the study’s implications before deciding whether to participate. In addition the study protocol was reviewed and approved by the competent authority Swissmedic.

All participants signed the Consent to Participate form.

The study has been registered on ClinicalTrials.gov (Identifier: NCT02157415).

### Study protocol

Inclusion criteria were hospitalized patients with urethral or suprapubic catheters for > 2 consecutive weeks, age ≥ 18 years, who were able to read, write and speak the local language who signed the informed consent. As this was the first study in humans using this device, a sustained pain sensation in the bladder was added as an inclusion criterion for the first five patients by the Competent Authority (Swissmedic) in order to ensure that pain was properly assessable. Exclusion criteria were urinary tract infection (UTI), hematuria, fever, surgical intervention to the genito-urinary tract within the last 6 months, any other catheter irrigation (concurrently or 1 week prior to the study), known allergy or sensitivity to any of the ingredients in the device, pregnancy or lactation, simultaneous participation in another interventional trial, and administration of any of the following medications 4 weeks prior to study entry, unless they have been used at a stable regimen: antibiotics, antipyretics, antihistamines, medications susceptible to cause an autonomic response or to mask an allergic reaction. At the first day, irrigation with 100 mL NaCl 0.9% solution was performed. If any significant changes in any of the primary outcomes are observed within 15 min following irrigation with saline, the subject was excluded from the study. All remaining participants were treated with the investigational device for five consecutive study days. Catheters were rinsed by gravity feed with 100 mL of the solution once a day. Before and 5 and 15 min after each instillation, respectively, participants were assessed for vital signs (heart rate, blood pressure and temperature), skin reactions and other symptoms suggesting intolerance or sensitivity and/or allergic reaction (occurrence of spasms, flushing and sweating). The urine was tested by dipstick before irrigation and 15 min post-irrigation. In addition, pain was assessed in the first 5 subjects and any other subjects with sustained pain sensation in the bladder by a VAS score with a scale from 0 to 100 mm. In addition, all participants had a final treatment assessment 18 to 30 h after the last treatment.

A sample size of 50 intent-to-treat participants was envisaged based on feasibility considerations and was deemed to be sufficient to assess tolerability. In agreement with the notified body and based on the low recruitment and the good tolerability results, the study was stopped early after 33 of 50 envisaged patients were enrolled.

### Statistical analysis methods

Descriptive statistics were calculated for all recorded data. For nominal and ordinal variables, absolute and relative frequencies (percentages) were calculated. For quantitative analysis variables, mean, standard deviation, minimum, median, lower quartile, upper quartile, interquartile range, and maximum were calculated. Additionally, changes of continuous variables before versus after instillation with UT-PHMB were categorized and additionally evaluated as ordinal variables. Summary statistics for adverse events and adverse device effects were coded using MedDRA version 21.1 and frequencies were calculated based on Preferred Term (PT) and System Organ Class (SOC) levels.

## Results

Between August 2013 and May 2018, a total of 33 participants were enrolled in the study out of which 29 completed all study visits according to protocol. Four participants prematurely terminated the study; one person due to an adverse event possibly related to the medical device. Three of those four patients withdrew consent owing to pain in lower extremity after saline irrigation, post-irrigation bladder irritation on day 2 and abdominal cramps after the fourth irrigation, respectively. The fourth patient was excluded from the study by the responsible investigator due to an adverse event possibly related to the medical device (urinary infection).

### Patient demographics and baseline characteristics

As one of the participants was excluded due to pain in the lower extremities after irrigation with saline, 32 participants received at least one treatment with the active ingredient and were therefore included in the analysis. Twenty-four (75%) of these 32 participants were male and eight (25%) were female. The mean age of the patients was 59.3 ± 13.2 years. All included participants had undergone urethral (*n* = 10) or suprapubic (*n* = 22) catheterization more than 2 weeks before the screening visit. Six patients were catheterized for more than 60 months at the time of screening. The most frequent medical conditions leading to catheter insertion were multiple sclerosis (*n* = 10), or spinal cord injury (*n* = 12) (Table 1).

**Table 1:** Demographic data and underlying disorder

Male (n)	24
Female (n)	8
Age (years)	59.3 + 13.2
Duration of catheterization (months)	28.80 + 48.02
Underlying disease (n)	
Multiple Sclerosis	10
Spinal Cord lesion	15
ischemic stroke	2
central cord syndrome	1
Critical illness polyneuropathy	1
Guillan-Barré-Syndrome	1
others	2

### Safety parameters

There was no incidence of a serious adverse event in the study. A total of 28 adverse events (AEs) were experienced by 15 (46.88%) of the participants (Table 2). Of those 15 persons, eight persons experienced a single AE while seven patients experienced more than one AE.

**Table 2:** Frequency of adverse events

	*N* = 32					
	Not related			Related		
	Mild	Moderate	Severe	Mild	Moderate	Severe
	n (%)	n (%)	n (%)	n (%)	n (%)	n (%)
Abdominal pain	1 (3.1)	0 (0.0)	0 (0.0)	0 (0.0)	0 (0.0)	0 (0.0)
Diarrhoea	1 (3.1)	0 (0.0)	0 (0.0)	0 (0.0)	0 (0.0)	0 (0.0)
Pain	0 (0.0)	0 (0.0)	0 (0.0)	1 (3.1)	0 (0.0)	0 (0.0)
Pyrexia	0 (0.0)	1 (3.1)	0 (0.0)	1 (3.1)	0 (0.0)	0 (0.0)
Urinary tract infection	0 (0.0)	0 (0.0)	0 (0.0)	1 (3.1)	0 (0.0)	0 (0.0)
Fall	1 (3.1)	0 (0.0)	0 (0.0)	0 (0.0)	0 (0.0)	0 (0.0)
Muscle spasms	0 (0.0)	0 (0.0)	0 (0.0)	0 (0.0)	2 (6.3)	0 (0.0)
Bladder discomfort	0 (0.0)	0 (0.0)	0 (0.0)	1 (3.1)	0 (0.0)	0 (0.0)
Bladder irritation	0 (0.0)	0 (0.0)	0 (0.0)	1 (3.1)	0 (0.0)	0 (0.0)
Bladder pain	0 (0.0)	0 (0.0)	0 (0.0)	2 (6.3)	2 (6.3)	0 (0.0)
Bladder spasm	0 (0.0)	0 (0.0)	0 (0.0)	6 (18.8)	3 (9.4)	0 (0.0)
Urethral pain	0 (0.0)	0 (0.0)	0 (0.0)	0 (0.0)	1 (3.1)	0 (0.0)
Urinary incontinence	0 (0.0)	0 (0.0)	0 (0.0)	0 (0.0)	1 (3.1)	0 (0.0)
Cough	0 (0.0)	1 (3.1)	0 (0.0)	0 (0.0)	0 (0.0)	0 (0.0)
Hypertension	1 (3.1)	0 (0.0)	0 (0.0)	0 (0.0)	0 (0.0)	0 (0.0)

The intensities of all AEs were either mild (*n* = 17, 61%) or moderate (*n* = 11, 39%) in nature (Table 2). Most of the adverse events were isolated in occurrence (*n* = 21, 75%) while seven (*n* = 7, 25%) AEs were classified as intermittent. All but one AEs (96.4%) were resolved with no sequelae by the end of the study. Of the 27 AEs which were resolved, 23 required no intervention, 2 were administered concomitant medications and for 1 AE the study product was stopped. One AE (spasms) was not resolved by the end of the study. However, persistence of spasms experienced by the patient further than the time of the treatment were diagnosed by the PI to be related to an underlying disease and not to the device, and thus a follow-up was not applicable.

Among the 28 reported AEs, 22 (78.6%) were classified as Adverse Device Effects (ADEs). For these 22 ADEs, the association between the AEs and the medical device was classified as possible (*n* = 16; 72.7%), probable (*n* = 2; 9.1%) and certain (4; 18.2%). The ADEs were experienced by 10 patients, four of which experienced one ADE each and six more than one ADE. ADEs were related to renal and urinary disorders. None of the ADEs were related to urticaria, exanthema or other allergic reactions.

### Tolerability and other safety parameters

VAS scores recorded for all participants having sustained sensation in the bladder were either zero or low for all visits, instillations and time-points. Absolute changes in VAS scores for all patients, visits and irrigation-types were less than 20 mm and, therefore, were not clinically relevant. No incidence of either flushing or sweating for any participant during any instillation was observed. Bladder spasms during instillation were reported in two cases, each at a single instillation. Four participants, who reported 9 incidences, reported them as moderate or mild. These spasms were experienced during a single instillation with UT-PHMB per patient and the patients did not reported any further incidences in the subsequent instillations. Additionally, no patient reported bladder spasms on the day of the final assessment.

**Fig. 1:**
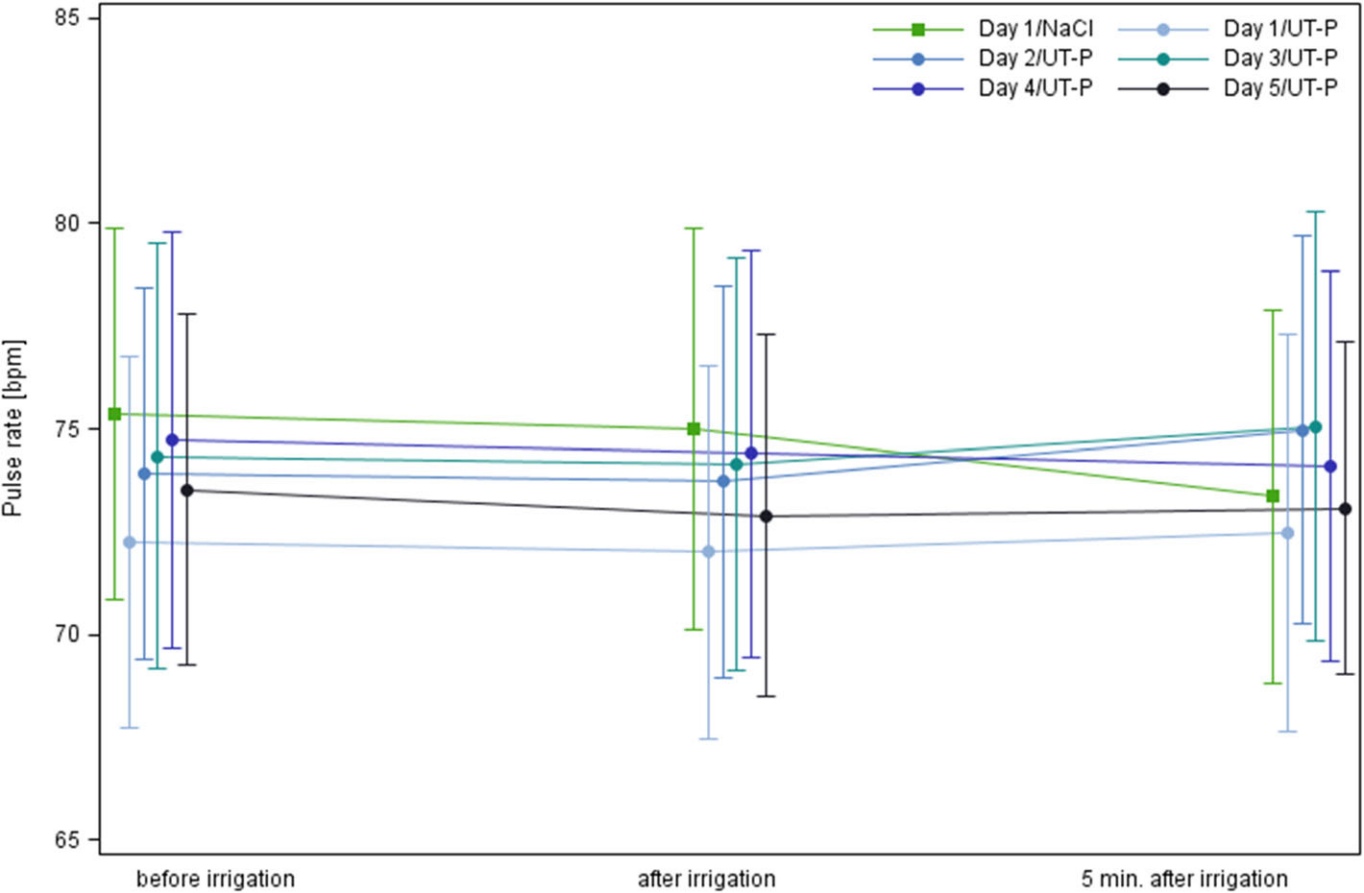
Pulse rate before, immediately after and 5 min after irrigation

Mean pulse rates of all study participants measured before, immediately after and 5 min after irrigation for any instillation with either saline solution or UT-PHMB did not change by more than 3 beats per minute (Fig. 1).

Absolute change in body temperature before and 15 min after irrigation was less than 2 °C across all visits and instillations for all but one participant who showed an increase of 2.1 °C in body temperature after the UT-PHMB instillation on study day 2 but did not show any signs of infection or any other signs of discomfort. Mean changes in body temperature did not exceed 0.12 °C in any comparison of pre - and post - irrigation temperatures.

**Fig. 2:**
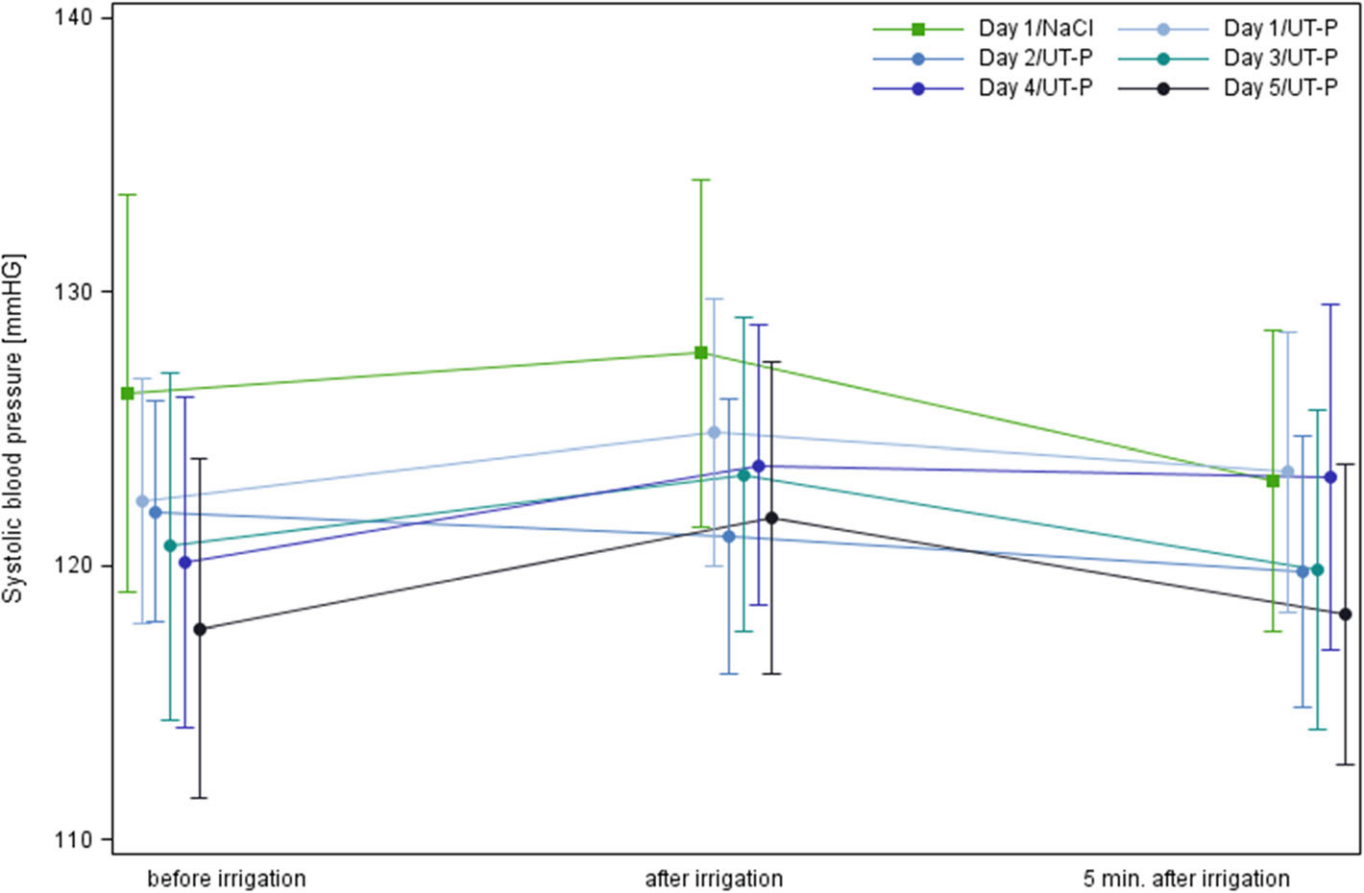
Systolic blood pressure before, immediately after and 5 min after irrigation

Mean systolic blood pressure during days 1–5 measured at different time points differed from the mean systolic blood pressure at screening (120.1 ± 17.5) by a maximum of 8 mmHg (Fig. 2). Clinically relevant changes in blood pressure were recorded for 3 patients. The blood pressure of one participants decreased by 53 mmHg after one irrigation while an increase was observed for the other two patients (40 mmHg and 70 mmHg, respectively) after irrigation with the device.

## Discussion

Results of the safety and tolerability analysis of the 32 patients included in this trial indicate that multiple instillations with polihexanide were safe and well tolerated. Although there were rare cases of discomfort and adverse events, these were temporary and did not persist throughout the course of the study. None of the patients experienced allergic reactions to the contents of the medical device. Furthermore, among the vital signs of patients monitored during the study, neither changes in pulse rate before and after instillation with the medicinal product nor in body temperature, blood pressure, pulse rate and VAS scores were clinically relevant for any participant. The overall low scores for all participants recorded over 5 instillations suggests that they experienced no noticeable pain during the instillations. In addition, no incidences of flushing or sweating during instillation were observed, and no participant experienced bladder spasms at each instillation. The three participants who did experience mild bladder spasms at all were afflicted by some form of spasticity (ischemic stroke, tetraplegia, multiple sclerosis), and muscle or bladder spasm in these patients can be induced by various stimuli, e.g. by mechanical stimulation of the bladder, through movement of the catheter and balloon [10]. Indeed, one of the four patients reported having experienced bladder spasms both after irrigation with saline solution and the tested solution, suggesting that the discomfort could have been induced by stimuli other than the medical device.

Colonization of urinary catheter with microorganisms is inevitable, and it is perpetuated by microorganisms-produced biofilm. Following insertion of a urinary catheter, a conditioning film made up of proteins, electrolytes, and other components of urine is deposited on the surface of the catheter. Microbes attach to this conditioning film and begin secreting polysaccharides that form the architectural structure of biofilm. Bacteria in biofilms are more difficult to treat than bacteriuria without the presence of a foreign body [11]. Under favorable conditions, organisms can detach from the biofilm and become free-floating in the urine, which can lead to symptomatic infection [12]. Another possible harmful consequence of bacteriuria and biofilm formation is the encrustation of the catheter which disturbs liquid drain. In the catheter the biofilm produces urease, which hydrolyzes urea in the patient’s urine to ammonium hydroxide. The resulting alkaline environment leads to precipitation of salts that then encrust and block the lumen of the catheter. About 50% of long-term catheterized patients develop such encrustations [6], which lead to urinary retention despite a catheter in place, with possibly fatal consequences especially in patients with neurogenic bladder dysfunction, e.g. autonomic dysreflexia [13].

To avoid encrustation, several strategies for catheter irrigation have been initiated. They either rely on mechanical cleansing of the bladder or on the use of citric acid to dissolve the crystals. Unfortunately, none of these strategies has been proven entirely successful, and the management of indwelling catheter remains to be a challenge. Alternatives, like triclosan-containing liquids for the inflating balloon, have demonstrated a certain effect, but these preliminary results have to be confirmed in further studies [14].

Treatment of chronic bacteriuria as the common reason for both UTI and encrustation is a challenge, and long-term eradication of bacteria from the urine in patients with long-term indwelling catheters has been proven to be virtually impossible, even with antibiotics [15]. Furthermore, the use of prophylactic antibiotics is discouraged because they may lead to the selection of resistant flora [16]. Antibiotic stewardship programs (ASP), which include prospective audits with intervention and feedback, and formulary restriction and pre-authorization, have been proven to be effective in reducing the unnecessary use of antibiotics [17]. One measure is the rational use of antibiotics which can be replaced by antiseptics. As local antiseptic treatment bladder instillations of chlorhexidine-containing solutions has been utilized [18]. Chlorhexidine diacetate, however, belongs to the first generation of this type of biguanide compound. It may cause some side effects (allergy, local irritation), and is known to produce a toxic degradation product (chloroaniline). In addition, bacterial resistance to chlorhexidine has been described [19].

The device assessed in the current study contains polihexanide 0.02%. Polihexanide has much higher tissue tolerability and is not degraded. It has been used for over 15 years as an antiseptic ingredient in wounds, and as a preservative agent in the ophthalmic field. It is a broad-spectrum biocide not only effective against Gram-positive and Gram-negative bacteria, but also against *Saccharomyces cerevisiae*, fungal and protozoal pathogens of infective keratitis, and against the enveloped virus HIV. The ecological database is still incomplete [9]. Polihexanide acts on the microorganisms by interacting with the negatively charged phospholipids of the bacterial membranes. Due to this non-specific action and the heterogeneity of polihexanide, the potential to induce resistance is very low. Furthermore, experimental studies have shown that polihexanide can accumulate in most biofilm matrices, which then become toxic to the resident microorganisms. In contrast polihexadine has only slight effect on the neutral lipids of human and animal cell membranes, thus providing a larger safety margin and risk-benefit ratio compared to other antimicrobial agents [20]. Based on the available data, polihexanide combines a broad antimicrobial spectrum with low toxicity, high tissue compatibility, low reported adsorption and good applicability [21]. Based on these favorable data, this clinical study has been performed to assess the safety and tolerability of polihexanide-containing solutions as routine rinsing and bacterial decolonization solution device for urinary catheters.

Possible drawbacks of the study are the low number of participants, which is less than the intended number, and the lack of data concerning the antimicrobial effect of the solution. This study, however, was designed to evaluate tolerability, as effectiveness testing requires a longer application period and could therefore only be assessed if the device is proven to be safe in clinical use. Although the foreseen number of participants has not been reached mainly due to the strict exclusion criteria, especially the use of antibiotics or antipyretics, the data from this study, combined with the clinical and preclinical data available for polihexanide, demonstrate sufficient evidence that the substance can safely be used for catheter irrigation. An initial in vitro study using a polihexanide solution in bladder catheters has already demonstrated bactericidal activity, leading to bacterial decolonization of the catheters [22]. Future studies should evaluate its clinical effectiveness in patients with long-term indwelling catheters. The proof of the safety and tolerability of the procedure is an important prerequisite for such a trial.

## Conclusion

The results of our trial indicate that multiple intravesical instillations with polihexanide were safe and well tolerated. Thus, further studies evaluating its clinical effectiveness in patients with long-term indwelling catheters seem to be feasible.

## Data Availability

The data that support the findings of this study are available from Braun, Medical AG, Sempach but restrictions apply to the availability of these data, which were used under license for the current study, and so are not publicly available. Data are however available from the authors upon reasonable request and with permission of [third party name]. The CRFs, tables and listings of all parameters evaluated in the course of the study are stored in the Trial Master File of B. Braun, Medical AG, Sempach, Switzerland. The data base and the data analysis were von GCP performed/provided by: GCP-Service Interna-tional Ltd. & Co. KG, Bremen, Germany.

## Abbreviations

AE: Adverse events
CAUTI: Catheter-associated urinary tract infections
UT-PHMB: Uro-Tainer® Polihexanide 0.02%
UTI: Urinary tract infection
